# The Complex Essential Oils Highly Control the Toxigenic Fungal Microbiome and Major Mycotoxins During Storage of Maize

**DOI:** 10.3389/fmicb.2019.01643

**Published:** 2019-07-16

**Authors:** Limin Wang, Bin Liu, Jing Jin, Longxue Ma, Xiaofeng Dai, Lin Pan, Yang Liu, Yueju Zhao, Fuguo Xing

**Affiliations:** ^1^Institute of Food Science and Technology, Chinese Academy of Agricultural Sciences/Key Laboratory of Agro-Products Quality and Safety Control in Storage and Transport Process, Ministry of Agriculture and Rural Affairs, Beijing, China; ^2^Shandong Quality Inspection Center for Medical Devices, Jinan, China

**Keywords:** fungal microbiome, mycobiome, mycotoxins, maize, ITS2 sequencing

## Abstract

The contamination of maize with fungi and subsequent mycotoxins is a pivotal and long-standing safety concern in the maize industry. In this study, the inhibitory effects of the complex essential oils (cinnamaldehyde, citral, eugenol, and menthol, 3:3:2:2, v/v) on fungal growth and mycotoxins production in stored maize were evaluated using traditional plate counting, internal transcribed spacer 2 (ITS2) sequencing and liquid chromatography–tandem mass spectrometry. Complex essential oils (0.02%) significantly (*p* < 0.05) reduced the total fungi counts and the content of aflatoxin B_1_, zearalenone, and deoxynivalenol in stored maize during 12 months of storage, and were more effective than propionic acid (0.2%). The fungal diversity of the control group was the highest with 113 operational taxonomic units. During storage of maize kernels, *Aspergillus*, *Fusarium*, *Wallemia*, *Sarocladium*, and *Penicillium* were main genera. At 0–6 months, the fungal diversity was high and *Fusarium* was predominant genus. However, at 7–11 months, the fungal diversity was low and *Aspergillus* was predominant genus. During the later stages of storage, the prevalence of *Aspergillus* in maize treated with essential oils was significantly lower than (*p* < 0.05) that observed in the propionic acid treated and control samples. The results of this study suggest that the complex essential oils may be employed successfully to control toxigenic fungi and subsequent contamination with mycotoxins in maize.

## Introduction

Maize, also known as corn, is a staple food in some countries or regions with greater production levels than wheat or rice. The America produces 40% of global maize yield, followed by China, Brazil, Argentina, Ukraine, India, and Mexico. In 2017, China produced 20.8% of the global maize harvest^1^ with 215.9 million tons^2^. Maize, being rich in B vitamins, thiamin, niacin, pantothenic acid (B_5_), and folate (USDA Nutrient Database), is an important source of food and feed (Zhu et al., 2016). However, mycotoxins contamination is a pivotal and long-standing concern in maize industry. The major mycotoxins contaminating maize include aflatoxins (AFs), zearalenone (ZEN), deoxynivalenol (DON), fumonisins (FUM), ochratoxins A (OTA), and alternariol. These toxins are predominantly biosynthesized by some fungal genera including *Aspergillus*, *Fusarium*, *Penicillium*, and *Alternaria* (Xing et al., 2017a). Among these mycotoxins, AFs are the most potent naturally occurring toxic and hepatocarcinogenic compounds and are estimated to bring about up to 28% of the global cases of hepatocellular carcinoma (HCC), the most common liver cancer (Wu, 2014; Xing et al., 2017b). Moreover, the simultaneous contamination of multi-mycotoxins in maize is very common (Aresta et al., 2003; Xing et al., 2017a).

To prevent, control, and eliminate harmful mycotoxins in our food chain, numerous strategies have been developed to control both mycotoxin production and fungal growth (Amaike and Keller, 2011; Xing et al., 2017b), or to eliminate and to degrade mycotoxins in foodstuff (Liang et al., 2015; Shcherbakova et al., 2015; Xing et al., 2017b). The actions include (i) preventing fungal infection on crops in field, for example by enhancing host resistance and using atoxigenic *Aspergillus parasiticus* and/or *Aspergillus flavus* strains or yeast (Chang et al., 2012; Xing et al., 2017b), (ii) controlling fungal growth during storage, by applying hypochlorite, organic acids, and natamycin (Panagou et al., 2005), and (iii) inhibiting mycotoxins biosynthesis, by applying natural products and microorganisms (Liang et al., 2015; Xing et al., 2017b).

Essential oils, as the complex mixtures of volatile aroma compounds, are extracted from living organisms or plant materials such as leaves, roots, flowers, buds, seeds, wood, fruits, twigs, and barks by distillation, pressing, solvent extraction, resin tapping, or wax embedding. Currently, more than 3000 essential oils have been isolated and widely used in perfumes, cosmetics, soaps, and other products, as food and drink flavorings, and for adding aromas to incense and household cleaning products (Vergis et al., 2015). Essential oils have proven antimicrobial, antioxidant, antiviral, antimycotic, antiparasitic, insecticidal, anti-inflammatory, and anticancer properties. In our previous study, 10 essential oils were assessed for their inhibitory effects on the growth of *A. flavus*, *Fusarium graminearum*, and *Fusarium verticillioides*, and mycotoxins production by fumigation and contact assays (Yuan et al., 2013). Cinnamaldehyde, citral, and eugenol were shown to be effective against *A. flavus* growth and AF production. Citral and eugenol could significantly inhibit *F. graminearum* growth and DON production. Citral, eugenol, and menthol were effective against *F. verticillioides* and FUM production (Yuan et al., 2013). In another study, we found that cinnamaldehyde, eugenol, and citral also could effectively inhibit fungal growth and OTA production by *Aspergillus ochraceus* (Hua et al., 2014). Furthermore, the mechanisms involved in the inhibitory properties of cinnamaldehyde, citral, and eugenol on fungal growth and mycotoxins biosynthesis were investigated using real-time PCR and RNA-Seq approaches. The results suggested that these essential oils inhibited mycotoxins biosynthesis by down-regulating the transcription levels of some regulator genes and pathway structural genes (Hua et al., 2014; Liang et al., 2015; Lv et al., 2018; Wang et al., 2018). The inhibitory effects of the above mentioned four essential oils on fungal growth (*Penicillium expansum*, *Aspergillus niger*, and *Aspergillus carbonarius*), and subsequent mycotoxin production has also been reported by other researchers (Bluma and Etcheverry, 2008; čvek et al., 2010; Passone et al., 2012).

The characterization of the microbiome on maize kernels is important in order to prevent and control mycotoxin production. However, it is often difficult and inaccurate to characterize the microbiome using traditional plate cultivation and colony counting techniques. Sequencing technologies have provided a high-throughput, high efficient, economic, and systematic method to analyze the microbial community in samples (Gregory Caporaso et al., 2011; Xing et al., 2016). Moreover, a revolution in the microbiome has been triggered by new-generation sequencing techniques through generating unprecedented numbers of sequences to detect extremely rare or low-abundance microorganisms (Begerow et al., 2010; Davey et al., 2012; Xing et al., 2016). These techniques have been used to characterize the microbiome present in our bodies (Costello et al., 2009; Grice et al., 2009; Ghannoum et al., 2010; Mar Rodríguez et al., 2015), grains (Ding et al., 2015; Xing et al., 2016), soils (Roesch et al., 2007; Lauber et al., 2009), and deep seas (Sogin et al., 2006). For fungal taxa assignment, the internal transcribed spacer 2 (ITS2) region of rDNA is widely used as a phylogenetic marker (Ding et al., 2015). Even though its potential lack of phylogenetic resolution, ITS2 region has been successfully used in comparative analysis of the moss-associated fungal communities (Davey et al., 2012) and the mycobiome in peanut kernels (Ding et al., 2015; Xing et al., 2016) and in wheat grains (Yuan et al., 2018).

According to the results of our previous studies and some literatures, the effect of complex essential oils, consisting of cinnamaldehyde, citral, eugenol, and menthol, on the microbiome and its variations in stored maize was elucidated using the barcoded Illumina paired-end sequencing (BIPES) technique in the present study. The production of the major mycotoxins in stored maize was also evaluated by liquid chromatography–tandem mass spectrometry (LC–MS/MS). The findings of this study might support the implementation of complex essential oils for the prevention and control of fungi and mycotoxins in grains during storage.

## Materials and Methods

### Essential Oils and Chemicals

Four natural essential oils, cinnamaldehyde (99%), citral (96%), eugenol (99%), and menthol (98%) were obtained from Jiangxi Xuesong Natural Medicinal Oil Co., Ltd., China. Propionic acid was purchased from Sinopharm Chemical Reagent Co., Ltd. The concentrations of these essential oils were confirmed in the laboratory. The complex essential oils consist of cinnamaldehyde, citral, eugenol, and menthol (3:3:2:2, v/v). The complex essential oils treatment was obtained by mixing essential oils and diatomite (1:1, v/m). The propionic acid treatment was obtained by mixing propionic acid and diatomite (1:1, v/m). Analytical standards for mycotoxins in acetonitrile, namely AF B_1_ (AFB_1_), ZEN, DON, OTA, Fumonisin B_1_ (FB_1_), and T-2 toxin were obtained from Sigma–Aldrich Chemicals (St. Louis, MO, United States). Chromatographic grade acetonitrile and methanol were obtained from Thermo Fisher Scientific (Waltham, MA, United States). Formic acid (purity > 98%) was obtained from Fluka (Buchs, Switzerland).

### Sample Preparation

Dent maize (*Zea mays* L. *indentata* Sturt.) kernels were obtained from Liaocheng City, Shandong Province, China, located in the Yellow River valley. After harvest and natural drying, the maize kernels were transported to our institute (Haidian District, Beijing, China) in 50-kg polypropylene woven bags (80 bags in total) in 3 days. These maize kernels were divided into three groups: control group (CK, untreated), essential oils treated group (CC), and propionic acid treated group (PA). For CC group, 160 g complex essential oils treatment was mixed with 400 kg maize kernels and the final concentration of complex essential oils was 0.02% (g/g). For PA group, 1,600 × *g* propionic acid treatment was mixed with 400 kg maize kernels and the final concentration of propionic acid was 0.2% (g/g). In the case of CK group, only 80 g diatomite was mixed with 400 kg maize kernels. The maize kernels were stored in small granaries from January 1, 2016 to December 31, 2016. During storage, the temperature of maize kernels, and the temperature and relative humidity in the small granaries were detected in real-time using an automatic supervisor system of temperature and humidity. Maize samples were collected once a month (0–12 months). Each group of samples was performed in triplicate. A total of 111 samples were collected.

### Determination of Moisture Content and Water Activity (*a*_w_)

The determination of the moisture content and *a*_w_ of maize kernels was performed according to the method described by Xing et al. (2017a) using an Aqualab 4TE Duo meter (Pullman, WA, United States).

### Determination of Colony-Forming Units (CFUs)

The maize kernels (25 g, about 80 kernels) were mixed with 100 ml of sterile deionized water, and then were shaken acutely for 30 min. The initial dilutions were obtained. The CFUs of fungi were determined by plating 100 μl of serial dilutions on Dichloran 18% Glycerol Agar (DG18) plates. The plates were incubated for 5 days at 30°C. After incubation, the fungal CFUs were calculated.

Speciation of isolated fungal strains was conducted as described by Madbouly et al. (2012). Purification of each isolate was done by subculturing on PDA plates. Each fungal isolate was identified to genus or species level according to colony morphology and microscopic examination, such as color, shape, size, structure and pigment of colony, structure and branching of mycelium, presence and shape of conidiophores, and sclerotia (Pitt and Hocking, 2009; Madbouly et al., 2012). As standard cultures, the reference strains were obtained from China General Microbiological Culture Collection Center (CGMCC, Beijing, China). The findings of conventional methods were further confirmed using the molecular method based on ITS sequencing as described by Xing et al. (2017a).

### DNA Extraction, PCR Analysis, Pyrosequencing, and Bioinformatics Analysis

The collected maize kernels (100 g) were washed with 100 ml sterile water. Then, the total fungi were collected and concentrated by vacuum-filtering the collected water samples with 0.22 μm membrane filters within 24 h. Total microbiome genomic DNA extraction, PCR analysis, pyrosequencing, and bioinformatics were performed according to the methods of Ding et al. (2015) and Xing et al. (2016). The fungal ITS2 fragments were amplified using the barcoded primers ITS2F (5′-GCATCGATGAAGAACGCAGC-3′) and ITS2R (5′-TCCTCCGCTTATTGATATGC-3′) according to the protocol described by Xing et al. (2016). Sequencing was subsequently conducted on an Illumina Miseq platform by Novogene (Beijing, China). The raw ITS2-Seq data of fungi discussed in this work have been deposited in the NCBI Sequence Read Archive under the accession number of PRJNA542593.

### Determination of Mycotoxins in Stored Maize Using LC–MS/MS

The contamination levels of major mycotoxins were determined as previous reports with minor modifications (Yibadatihan et al., 2014). In this experiment, 100 g of maize from each sample was ground to obtain maize meal. Maize meal (5 g) was mixed with 20 ml of extraction solvent of acetonitrile:water (84:16, v/v) by shaking on a shaker for 60 min. The mixture was filtered through double-layer fast qualitative filter paper, and then 8 ml of the defatted extract was purified with a MycoSpin 400 multi-mycotoxins column (Romer Labs, Newark, DE, United States) according to the manufacturer’s instructions. After purifying, 4 ml of extract was transferred into a 10 ml centrifuge tube and evaporated to dryness at 50°C under a gentle steam of nitrogen gas (Xing et al., 2017a). Then, 1 ml of diluting solution (Methanol:10 mmol/L ammonium acetate in water, 1:1, v/v) was added into the tube and mixed well to dissolve the residue. The final extract solution was collected in 2 ml glass tubes for LC–MS/MS quantitative analysis after passing through a 0.22-μm hydrophobic filter.

The LC–MS/MS equipment consisted of a 1260-series LC coupled to a 6420 Triple Quadystem mass spectrometer (Agilent Technologies, Santa Clara, CA, United States). An Agilent Proshell 120 SB-C18 (100 mm × 2.1 mm, 1.8 μm) was used as analytical column. The gradient elution solvent was A (0.1% formic acid in water) and B (0.1% acetonitrile in water) at a flow rate 0.2 ml/min. The initial composition was 70% A held for 1 min, followed by 30% A for 5 min, 10% A for 10 min, 70% A for 11 min, and 70% A for 20 min. The injection volume of the sample was 2 μl.

The electrospray ionization (ESI) analysis in PI mode was performed. MS parameters for the analysis were the following: gas temperature 350°C, gas proof curtain 350°C, gas flow (N_2_) 6 L/min, nebulizer pressure 40 psi, and ionization voltage 4 kV. To increase sensitivity and selectivity, data acquisition was operated in multiple reaction monitoring (MRM) mode on the protonated molecule for AFB_1_, ZEN, DON, FB_1_, OTA, and T-2 toxin.

### Statistics

The total fungi counts and mycotoxins concentrations in stored maize were expressed as mean ± relative standard deviation (RSD). Statistical significance was evaluated using the one-way analysis of variance (ANOVA) for multiple comparisons (SPSS20.0 Software). *P-*value < 0.05 was considered statistically significant.

## Results

### The Complex Essential Oils Highly Reduced Fungi During Storage of Maize

**FIGURE 1:**
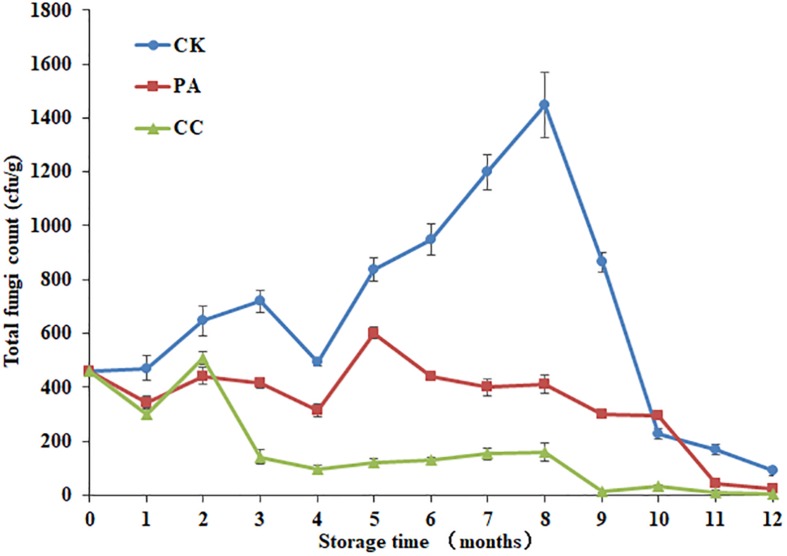
The effect of complex essential oils on the total fungal counts in stored maize. CK, control group; PA, propionic acid treated group; CC, complex essential oils treated group. The relative standard deviation of total fungi counts is represented with bars (*n* = 6).

As shown in Figure 1, both the compound essential oils and propionic acid highly reduced the total fungal counts in stored maize during 12 months storage. Moreover, the complex essential oils (0.02%) were more effective against fungi than propionic acid (0.2%). By the 8th month, the inhibition rates of complex essential oils and propionic acid were 89.0 and 71.7%, respectively. At the 5th month, the inhibition rates of complex essential oils and propionic acid were 49.5 and 16.4%, respectively. For CK group, the total fungal counts increased from 1 to 3 months, decreased at 4th month, and then increased from 4 to 8 months. By 8th month, the highest total fungal count was observed. However, the highest total fungal count was observed at 2nd and 5th month for CC group and PA group, respectively. It is important to note that the total fungal counts for CC group were below the 160 cfu/g during 3–12 months.

As shown in Table 1, *A. flavus*, *Aspergillus penicillioides*, *F. verticillioides*, *F. proliferatum*, and *Penicillium oxalicum* were the main fungi isolated on DG18. *F. graminearum*, *A. niger*, and *Fusarium culmorum* were also detected on maize kernels. In general, the complex essential oils and propionic acid highly reduced the CFUs of these fungi, especially during the late stage of storage. The inhibitory effects of the complex essential oils on fungal growth were stronger than those of propionic acid although the dosage of complex essential oils was only one-tenth that of propionic acid.

**TABLE 1:** The colony-forming units (CFUs) of main fungi in stored maize treated with complex essential oils (CC), propionic acid (PA), and untreated (CK).

**Fungi (CFUs/g)**	**Group**	**Storage time (months)**												
		**0**	**1**	**2**	**3**	**4**	**5**	**6**	**7**	**8**	**9**	**10**	**11**	**12**
*Aspergillus flavus*	CK	27.0±1.7	30.0±12^a^	24.0±4.2^a^	2.2±1.2^a^	15.5±3.4^a^	29.1±10.5^a^	159.8±15.7^a^	329.7±16.3^a^	573.3±27.9^a^	307.0±18.8^a^	30.3±8.8	15.0±8.2	21.0±4.2
	PA		5.0±0.8^b^	2.0±0.3^b^	1.2±0.7^b^	1.9±0.2^b^	20.0±0.4^b^	18.2±1.2^b^	28.4±3.2^b^	100.0±12.3^b^	3.3±1.2^b^	*ND*	*ND*	*ND*
	CC		4.0±0.9^b^	5.0±0.5^c^	*ND*	*ND*	10.4±1.9^c^	15.0±2.1^b^	13.1±3.5^c^	3.5±0.3^c^	*ND*	*ND*	*ND*	*ND*
*Aspergillus penicillioides*	CK	111.0±13.5	123.1±21.2^a^	231.1±15.4^a^	252.5±25.3^a^	149.1±17.7^a^	305.2±27.2^a^	245.2±14.5^a^	200.2±9.1^a^	260.0±13.4^a^	216.5±13.3^a^	65.0±7.3^a^	63.5±5.2^a^	19.0±3.2^a^
	PA		169.2±6.7^b^	235.0±10.8^a^	155.0±13.4^b^	208.0±12.1^b^	297.8±10.1^a^	90.0±7.7^b^	79.5±2.3^b^	10.0±3.3^b^	163.0±14.2^b^	195.0±21.4^b^	30.0±2.1^b^	18.0±1.5^a^
	CC		168.0±4.5^b^	263.0±9.3^b^	55.0±7.2^c^	40.0±5.9^c^	30.0±9.3^b^	41.0±2.3^c^	2.3±0.1^c^	19.1±0.9^c^	3.1±0.7^c^	20.0±4.8^c^	5.0±0.8^c^	1.3±0.2^c^
*Fusarium verticillioides*	CK	54.0±10.8	84.0±17.2^a^	127.0±21.2^a^	142.0±15.8^a^	50.0±8.3	19.72±11.3^a^	61.7±7.5^a^	75.4±10.5^a^	70.0±0.5	43.2±5.3	15.6±3.7	*ND*	*ND*
	PA		10.0±1.1^b^	16.0±5.3^b^	8.0±3.3^b^	*ND*	22.96±2.3^a^	16.9±5.2^b^	10.3±3.0^b^	*ND*	*ND*	*ND*	*ND*	*ND*
	CC		35.0±3.3^c^	12.0±2.2^c^	15.6±1.5^c^	*ND*	0.65±0.6^b^	3.3±0.8^c^	*ND*	*ND*	*ND*	*ND*	*ND*	*ND*
*Fusarium proliferatum*	CK	45.7±12.8	28.3±5.5^a^	42.5±7.2^a^	43.2±5.3^a^	17.3±2.4	6.4±2.9^a^	21.8±2.8^a^	26.3±3.2^a^	23.8±1.6	13.9±1.8	*ND*	*ND*	*ND*
	PA		3.9±0.4^b^	5.3±1.8^b^	2.7±1.2^b^	*ND*	7.3±0.8^a^	5.6±1.4^b^	3.5±2.1^b^	*ND*	*ND*	*ND*	*ND*	*ND*
	CC		11.5±2.8^c^	4.2±0.8^b^	5.2±0.4^c^	*ND*	*ND*	*ND*	*ND*	*ND*	*ND*	*ND*	*ND*	*ND*
*Penicillium oxalicum*	CK	245.0±15.4	120.0±10.2^a^	251.0±9.2^a^	293.1±19.3^a^	268.2±14.5^a^	294.1±10.3^a^	255.0±5.2^a^	300.9±13.2^a^	398.3±21.2^a^	243.0±42.5^a^	109.6±13.4^a^	69.5±14.3^a^	25.4±6.4^a^
	PA		150.0±10.8^b^	171.0±3.4^b^	197.4±7.2^b^	102.8±12.3^b^	252.3±15.6^b^	283.3±20.4^b^	254.5±12.4^b^	186.7±18.8^b^	103.6±9.4^b^	76.6±7.4^b^	9.3±3.2^b^	4.7±1.1^b^
	CC		70.0±2.2^c^	189.0±12.2^c^	64.0±8.3^c^	49.5±5.6^c^	75.3±8.2^c^	50.0±6.4^c^	84.3±10.3^c^	95.8±14.4^c^	7.0±1.2^c^	4.4±0.8^c^	1.7±0.3^c^	3.3±0.5^b^
others	CK	23.1±2.1	114.2±15.5^a^	13.0±2.3^a^	29.7±3.3^a^	11.9±5.4^a^	191.0±17.3^a^	228.4±14.8^a^	292.5±12.3^a^	147.6±17.4^a^	54.9±12.1^a^	7.0±0.4^a^	20.8±3.2^a^	23.4±5.3^a^
	PA		10.4±1.3^b^	17.5±3.4^b^	54.2±4.4^b^	3.3±0.8^b^	8.9±0.4^b^	31.6±2.4^b^	16.4±1.4^b^	113.3±11.2^b^	30.6±5.7^b^	21.6±0.7^b^	1.3±0.3^b^	2.2±0.5^b^
	CC		24.6±1.6^c^	41.0±1.4^c^	6.6±0.5^c^	6.4±0.4^c^	5.2±1.2^c^	22.3±2.1^c^	0.4±0.1^c^	41.5±5.9^c^	3.0±0.9^c^	7.8±1.2^a^	1.3±0.5^b^	1.0±0.2^c^

^*^ND, not detected. Different lowercase letters in each column indicate a significant (*P* < 0.05). CK, control group; PA, propionic acid treated group; CC, essential oils treated group.

### The Effects of Complex Essential Oils on the Main Mycotoxins Production During Storage of Maize

The mycotoxin levels in stored maize are presented in Table 2. AFB_1_, ZEN, DON, FB_1_, OTA, and T-2 were detected in the original sample. AFB_1_, ZEN, and DON were main mycotoxins in maize kernels during storage, while OTA and T-2 were only detected in the 1st month. FB_1_ was detected at the start of 5 months of storage, while it was undetectable at 7–12 months. The contents of DON were the highest of the three main mycotoxins. For CK, PA, and CC group, the levels of DON were 158.0–640.2, 34.7–268.7, and 31.5–227.2 μg/kg, respectively. Overall, the contents of AFB_1_, ZEN, and DON in PA and CC groups’ samples were significantly lower than those in CK samples. Furthermore, the levels of AFB_1_ and ZEN in CC samples were lower than those in PA samples. In the CC maize kernels, the content of AFB_1_ was ≤5.4 μg/kg during storage, and levels were undetectable during the last 3 months. These results suggest that both the complex essential oils and propionic acid can highly inhibit the production of AFB_1_, ZEN, DON, and FB_1_. The inhibitory effects of the complex essential oils on AFB_1_ and ZEN production were stronger than those of propionic acid although the dosage of complex essential oils was only one-tenth that of propionic acid.

**TABLE 2:** The concentration of main mycotoxins in stored maize treated with complex essential oils (CC), PA, and untreated (CK).

**Mycotoxins (μg/kg)**	**Group**	**Storage time (months)**												
		**0**	**1**	**2**	**3**	**4**	**5**	**6**	**7**	**8**	**9**	**10**	**11**	**12**
AFB_1_	CK	3.0±0.1	9.7±0.2^a^	16.6±1.2^a^	14.1±1.8^a^	3.3±2.7^a^	3.9±2.5^a^	10.0±0.1^a^	17.3±0.9^a^	21.1±3.5^a^	13.7±2.4^a^	11.6±0.7^a^	15.4±1.3	13.1±1.0^a^
	PA		6.8±0.5^b^	6.8±0.9^b^	2.7±0.5^b^	1.7±0.2^b^	1.0±0.1^b^	3.3±0.4^b^	2.6±0.6^b^	1.5±0.3^c^	0.7±0.2^b^	1.3±0.3^b^	*ND*	0.6±0.1^b^
	CC		5.4±0.4^c^	3.5±0.8^c^	1.5±0.6^c^	0.5±0.1^c^	0.5±0.1^c^	0.4±0.2^c^	2.3±0.4^b^	2.0±0.8^b^	0.5±0.1^c^	*ND*	*ND*	*ND*
ZEN	CK	12.0±2.1	12.7±1.5	13.8±0.5^a^	14.7±0.3^a^	14.3±1.9^a^	4.9±2.1^b^	10.4±0.4^a^	11.3±1.3^a^	7.4±0.5^a^	12.5±1.4^a^	5.4±0.9^a^	6.8±0.5^a^	13.8±1.5^a^
	PA		11.8±1.4	12.5±1.0^a^	8.0±0.5^b^	14.1±0.5^a^	11.0±3.5^a^	5.1±1.0^b^	7.9±0.5^b^	4.1±0.2^b^	3.9±1.8^b^	3.8±1.0^b^	2.3±0.2^b^	1.4±0.1^b^
	CC		11.8±2.0	8.4±0.8^b^	4.6±0.7^c^	2.1±1.5^b^	4.7±0.5^b^	3.4±0.4^c^	4.2±0.1^c^	2.7±0.3^c^	1.5±0.2^c^	1.1±0.3^c^	1.0±0.1^c^	0.8±0.1^c^
DON	CK	100.3±2.0	158.0±5.4^a^	270.1±2.8^a^	313.0±15.2^a^	330.3±16.8^a^	184.8±12.6^a^	412.5±12.8^a^	527.2±15.3^a^	640.2±10.3^a^	635.5±31.2^a^	539.4±18.3^a^	281.7±15.1^a^	383.0±12.4^a^
	PA		159.9±3.5^a^	268.7±5.9^a^	191.1±10.3^b^	230.2±10.2^b^	111.9±5.4^b^	204.3±2.2^b^	156.8±20.0^c^	77.0±3.2^c^	60.5±10.3^c^	34.7±5.2^b^	102.4±2.3^c^	72.3±5.2^c^
	CC		133.4±1.2^b^	171.1±3.2^b^	114.6±15.6^c^	227.2±1.5^b^	176.2±7.2^a^	149.8±10.3^c^	191.5±12.2^b^	217.4±21.3^b^	142.2±19.4^b^	31.5±2.3^b^	143.3±7.4^b^	86.3±9.4^b^
FB_1_	CK	140.5±5.3	70.5±5.6^a^	82.2±10.1^a^	32.1±0.5^a^	102.6±2.3^a^	16.5±1.0^a^	*ND*	*ND*	*ND*	*ND*	*ND*	*ND*	*ND*
	PA		68.7±2.0^a^	49.0±11.3^b^	24.6±0.3^b^	31.5±0.5^b^	10.2±3.1^b^	*ND*	*ND*	*ND*	*ND*	*ND*	*ND*	*ND*
	CC		51.4±9.3^b^	34.1±9.2^c^	22.3±0.1^b^	15.6±1.0^c^	5.3±1.6^c^	*ND*	*ND*	*ND*	*ND*	*ND*	*ND*	*ND*
OTA	CK	1.3±0.7	1.5±1.0	*ND*	*ND*	*ND*	*ND*	*ND*	*ND*	*ND*	*ND*	*ND*	*ND*	*ND*
	PA		1.0±0.5	*ND*	*ND*	*ND*	*ND*	*ND*	*ND*	*ND*	*ND*	*ND*	*ND*	*ND*
	CC		1.5±1.0	*ND*	*ND*	*ND*	*ND*	*ND*	*ND*	*ND*	*ND*	*ND*	*ND*	*ND*
T-2	CK	0.5±0.1	0.5±0.1	*ND*	*ND*	*ND*	*ND*	*ND*	*ND*	*ND*	*ND*	*ND*	*ND*	*ND*
	PA		0.5±0.1	*ND*	*ND*	*ND*	*ND*	*ND*	*ND*	*ND*	*ND*	*ND*	*ND*	*ND*
	CC		0.5±0.0	*ND*	*ND*	*ND*	*ND*	*ND*	*ND*	*ND*	*ND*	*ND*	*ND*	*ND*

^*^ND, not detected. Different lowercase letters in each column indicate a significant (*P* < 0.05). AFB_1_, aflatoxin B_1_; ZEN, zearalenone; DON, deoxynivalenol; FB_1_, fumonisin B_1_; OTA, ochratoxin A; T-2 = T-2 toxin; CK, control group; PA, propionic acid treated group; CC, essential oils treated group.

### Data Characteristics of ITS2 Sequencing

Out of 111 maize samples stored in Beijing, 102 samples were successfully amplified and sequenced. Other nine samples including the maize kernels collected from CK, PA, and CC group at 12th month (triplicate for each group) were not amplified due to the less total fungal counts (Figure 1). In CK group, the average number of raw tags generated per sample was 57,546, then 55,749 were retained after filtering steps, and 53,058 tags were subsequently clustered into different OTUs (Table 3). In PA group, the average numbers of raw tags, clean tags, and effective tags were 56,431, 54,553, and 52,132, respectively. In CC group, the average numbers of raw tags, clean tags, and effective tags were 55,809, 53,993, and 51,411, respectively.

**TABLE 3:** Summary of pyrosequencing analysis in stored maize treated with complex essential oils (CC), PA, and untreated (CK).

**Storage time (month)**	**CK**		**PA**		**CC**	
	
	**Effective tags**	**Average length (bp)**	**Effective tags**	**Average length (bp)**	**Effective tags**	**Average length (bp)**
0	56,277	317	56,277	317	56,277	317
1	56,277	317	58,023	315	53,193	311
2	54,382	310	55,662	312	54,855	313
3	52,642	319	46,426	314	51,282	316
4	54,740	314	55,925	314	52,103	311
5	53,074	314	52,520	305	58,720	312
6	52,523	315	52,344	311	49,729	314
7	52,530	314	49,404	309	49,880	310
8	52,017	313	54,339	309	51,563	319
9	50,442	331	47,694	321	45,168	337
10	47,623	331	50,781	321	47,705	328
11	54,166	341	46,186	333	46,454	349
12	/	/	/	/	/	/
Average	53,058	320	52,132	315	51,411	320

*CK, control group; PA, propionic acid treated group; CC, essential oils treated group; bp, base pair.*

### Fungal Diversity in Stored Maize Kernels

**FIGURE 2:**
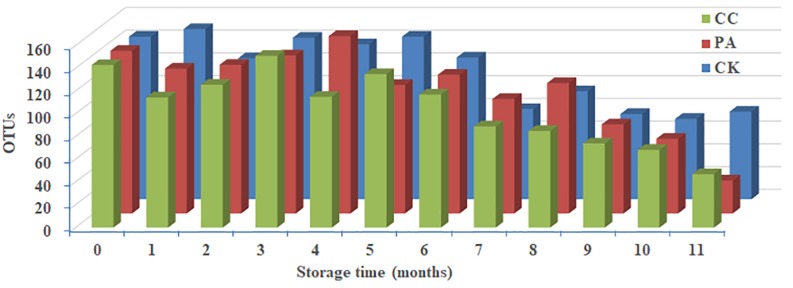
Predicted number of operational taxonomic units (OTUs) per sample in stored maize. CK, control group; PA, propionic acid group; CC, complex essential oils group.

Significant change in per-sample OTU richness was observed based on storage time and maize kernels in different groups (Figure 2). For CK group, 53,058 effective tags per sample were clustered into 76 OTUs. The average OTUs number in CK maize kernels was 113 with a range of 71–150. For PA group, 52,132 effective tags per sample were clustered into 166 OTUs, and the average number of OTUs per sample was 110 with a range of 29–156. In the case of CC group, 51,411 effective tags per sample were clustered into 93 OTUs, and the average number of OTUs per sample was 105 with a range of 47–151. As shown in Figure 2, the fungal diversities fluctuated during the storage. In principle, the fungal diversity was lower at the later stage of storage (7–11 months) than during the early stages (0–6 months).

The nine most abundant genera and main fungal species in these genera detected in stored maize kernels were shown in Table 4. Ascomycota was dominant in the CK, PA, and CC groups, with 86.79, 95.32, and 84.67% of reads belonging to the phylum, respectively. Among the Ascomycota, *Aspergillus*, *Talaromyces*, *Cercospora*, *Meyerozyma*, *Fusarium*, and *Sarocladium* were main genera observed. Basidiomycota was another dominant phylum detected. In Basidiomycota, *Wallemia* was the only main genera observed. The predominant species in *Aspergillus* were *A. flavus*, *A. penicillioides*, and *A. niger*. In *Talaromyces*, *Cercospora*, *Fusarium*, *Sarocladium*, and *Wallemia*, the predominant species were *Talaromyces funiculosus*, *Cerospora sojina*, *F. verticillioides* and *Fusarium proliferatum*, *Sarocladium zeae*, and *Wallemia sebi*, respectively. In the PA group, the relative abundance of *W. sebi* was obviously lower than that in the CK and CC groups, suggesting that propionic acid could highly inhibit the growth and proliferation of *W. sebi*.

**TABLE 4:** The nine most abundant genera detected in stored maize kernels.

	**The average percent of reads during the 12 months storage (%)**		
**Taxonomic affinity**	**CK**	**PA**	**CC**
Ascomycota	86.79	95.32	84.67
Eurotiomycetes	37.95	37.52	34.04
*Aspergillus*	31.68	30.81	27.88
*A. flavus*	7.78	9.27	9.13
*A. penicillioides*	17.48	18.32	14.87
*A. niger*	6.42	3.23	3.88
*Talaromyces*	4.49	4.18	3.93
*T. funiculosus*	4.49	4.18	3.93
*Penicillium*	1.78	2.53	2.26
*P. citrinum*	0.48	0.57	0.57
*P. oxalicum*	1.30	1.96	1.69
Dothideomycetes	3.22	4.25	3.31
*Cercospora*	3.06	3.77	3.18
*C. sojina*	3.06	3.77	3.18
Saccharomycetes	3.98	4.09	3.18
*Meyerozyma*	3.57	3.40	2.75
Sordariomycetes	41.64	49.45	44.12
*Fusarium*	34.11	40.88	36.42
*F. verticillioides*	30.67	37.29	33.73
*F. proliferatum*	3.04	3.00	2.24
*F. intricans*	0.40	0.59	0.45
*Sarocladium*	5.48	7.30	6.32
*S. zeae*	5.48	7.30	6.32
*Trichoderma*	2.05	1.27	1.37
*T. harzianum*	1.81	1.10	1.19
*T. asperellum*	0.24	0.16	0.19
Basidiomycota	13.11	4.65	15.32
Wallemiomycetes	13.11	4.65	15.32
*Wallemia*	13.11	4.65	15.32
*W. sebi*	13.11	4.65	15.32

*CK, control group; PA, propionic acid treated group; CC, essential oils treated group; bp, base pair.*

### The Effects of Complex Essential Oils on Fungal Community Variation in Genus Level Across Storage Time

**FIGURE 3:**
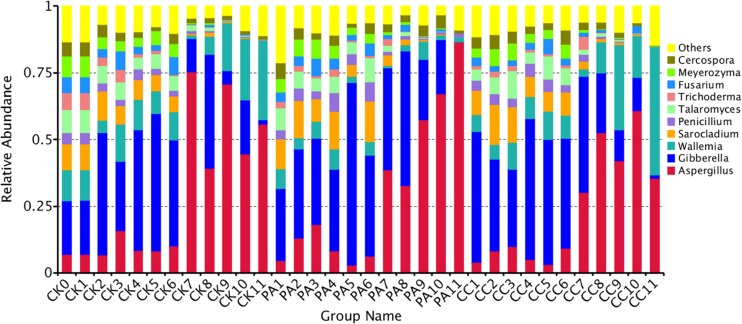
Overall distribution of fungi at genus level in stored maize. CK0, raw maize; CK1–CK11, stored maize in control group at 1–11 months; PA1–PA11, stored maize treated with propionic acid at 1–11 months; CC1–11, stored maize treated with complex essential oils.

The fungal community variation per sample in genus level based on treatment type and storage time was shown in Figure 3. The *a*_w_ values of maize kernels were in the range of 0.57–0.68 during storage. At the early stages of storage (0–6 months), *Fusarium* were predominant genera. However, at the later stages of storage (7–11 months), *Aspergillus* became the predominant genus. The relative abundance of *Aspergillus* sp. in CK, PA, and CC samples increased from 9.05, 8.92, and 6.62% to 57.14, 56.51, and 44.23%, respectively. The relative abundance of *Sarocladium*, *Penicillium*, *Talaromyces*, *Trichoderma*, *Cercospora*, and *Meyerozyma* in all three groups obviously decreased in the later stages of storage. For CK and CC group, the relative abundance of *Wallemia* increased in the later stages of storage. However, for PA group, the relative abundance of *Wallemia* decreased from 5.42 to 2.68% in the later stages of storage. In the later stages of storage, the relative abundance of *Aspergillus* in CC group was lower than that in CK and PA groups. These results suggested that the complex essential oils were more effective against *Aspergillus* sp. compared with propionic acid while propionic acid was more effective against *Wallemia* sp.

### Changes in Mycobiome Associated With Storage Time and Different Treatment

**FIGURE 4:**
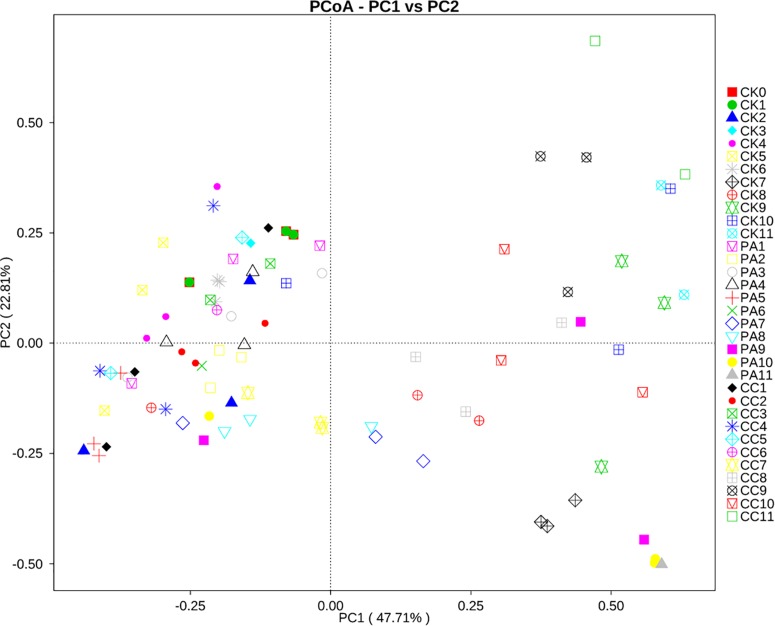
Principal coordinate analysis (PCoA) of distribution of fungi in stored maize. CK0, raw maize; CK1–CK11, stored maize in control group at 1–11 months; PA1–PA11, stored maize treated with propionic acid at 1–11 months; CC1–11, stored maize treated with complex essential oils.

To investigate the correlation between the changes in mycobiome and the storage time or the different treatment, principal coordinate analysis (PCoA) was performed. As shown in Figure 4, the maize kernels stored for 1–6 months were clustered together. However, the maize kernels stored for 7–11 months were scattered. In the case of the CK group, all maize kernels stored for 1–6 months were clustered in the left with PCoA case scores (Bray–Curtis) less than zero, and the maize kernels stored for 7–11 months were clustered in the right with scores more than zero, with the exception of one sample (CK10, from 10 months). For PA group, all maize kernels stored for 1–6 months clustered in the left and most maize kernels stored for 7–11 months clustered in the right, except for a small number of samples including one PA7, two PA8, one PA9, and one PA10. For CC group, all maize kernels stored for 1–7 months clustered in the left, and all maize kernels stored for 8–11 months clustered in the right. These results suggest a trend in association between the maize mycobiome and storage time or treatment.

## Discussion

The contamination with fungi and subsequent mycotoxins is regarded to be one of the world’s most severe food safety concerns (Williams et al., 2004). To eliminate the contamination, a number of chemicals and natural products have been evaluated to either prevent fungal growth or inhibit mycotoxin biosynthesis (Lv et al., 2018). Moreover, there is now a trend toward the use of innovative methods with natural or “green” character; for example, the application of essential oils in food industries as alternatives to chemical fungicides (Vergis et al., 2015). In the present study, the results confirmed that the complex essential oils (0.02%) highly reduced the total fungal counts and were more effective than propionic acid (0.2%) against fungi in stored maize. At the 8th month of storage, the total fungal counts of the CK group were the highest, and the inhibition rates of essential oils and propionic acid were 89.0 and 71.7%, respectively. Similarly, both the complex essential oils and propionic acid could significantly reduce the levels of the main mycotoxins including AFB_1_, ZEN, DON, and FB_1_. The reduction rates of mycotoxins in maize treated with the complex essential oils were higher than those in maize treated with propionic acid although the dosage of essential oils was only one-tenth that of propionic acid. Our results confirm previous findings (Velluti et al., 2003, 2004; Marín et al., 2004; Bluma and Etcheverry, 2008; čvek et al., 2010; Passone et al., 2012; Yuan et al., 2013), suggesting that essential oils represent good candidates in controlling of toxigenic fungi and subsequent mycotoxins in stored grains.

In general, *Aspergillus*, *Cercospora*, *Fusarium*, *Meyerozyma*, *Penicillium*, *Sarocladium*, *Talaromyces*, *Trichoderma*, and *Wallemia* were main genera in stored maize kernels, due to the more adaptation of them to the low *a*_w_ and the substrate, especially during storage (Mudili et al., 2014; Xing et al., 2017a, 2018). The occurrence of *Aspergillus*, *Fusarium*, *Penicillium*, and *Trichoderma* in maize has also been reported in other studies. In 2014, the mycoflora in pre- and post-nature drying maize from North China Plain was evaluated using a culture-based approach and it was found that *Fusarium*, *Aspergillus*, *Alternaria*, and *Trichoderma* were main genera. At species level, *F. verticillioides* (24.77%) and *F. graminearum* (15.08%) were predominant species, followed by *A. niger* (7.51%) and *A. flavus* (4.93%) (Xing et al., 2017a). Xing et al. (2018) investigated the mycobiota of 11 maize samples from the Gansu Province of China with storage times ranging from 6 months to 12 years using a culture-based approach. They found that *A. niger* was present in all samples and dominated the fungal species with a relative abundance of 36–100%, and species of *Fusarium* (9–40%) and *Penicillium* (9–20%) genera were also frequently isolated. Mudili et al. (2014) investigated mold incidence in freshly harvested maize samples from southern India and found that these samples were highly contaminated with toxigenic fungi of *Aspergillus*, *Fusarium*, and *Penicillium* genera. Chauhan et al. (2016) found the massive prevalence of *Aspergillus* species (75%) followed by *Fusarium* (11%), *Penicillium* (8%), and *Trichoderma* (6%) in maize collected from Gedeo Zone of Ethiopia as characterized by biochemical and sporulation properties. Alborch et al. (2012) reported that *Aspergillus* spp. and *Penicillium* spp. were the predominant mycobiota in maize flours. In popcorn kernels, *Aspergillus* spp., Mucorales, *Fusarium* spp., and *Penicillium* spp. were the most frequent species. These results indicated that *Aspergillus*, *Fusarium*, and *Penicillium* were the predominant mycobiota in maize using culture-based approaches. In the present study, there was a greater variety of genera observed using an ITS2 sequencing approach compared to the traditional culture-based approach.

During storage, the fungal diversity of maize samples obviously decreased across the storage time. At the beginning of storage, the relative abundances of top 10 genera were similar (Figure 3). Then at the early stages of storage (1–6 months), *Fusarium* (including *Gibberella*) was the predominant genus in all three groups. However, during the later stages of storage (7–11 months), *Aspergillus* became the predominant genus in all three groups. The highest values in the CK, PA, and CC groups were 75.3, 86.5, and 60.9%, and were observed at the 7th, 11th, and 10th months, respectively. In particular, the relative abundances of *Aspergillus* in maize kernels of PA group increased from 8 to 11 months and reached the highest value 86.5%. The result was similar with the findings of our previous study in stored peanut kernels (Ding et al., 2015). During storage, the relative abundance of *Aspergillus* in peanut kernels was higher at 7–12 months than the first 6 months. Similarly, in order to reveal the inter-annual variability in fungal communities, Xing et al. (2018) examined maize kernels with the storage times ranging from 6 months to 12 years and found that *Aspergillus* (*A. niger*) predominated the fungal microbiota and surpassed *Fusarium* (*F. verticillioides* and *F. proliferatum*), the most prevalent fungal genus in maize kernels.

In the early stages of storage (1–6 months), the average relative abundances of *Aspergillus* in maize kernels from the CK, PA, and CC groups were 9.4, 8.9, and 6.6%, respectively. However, the values in CK, PA, and CC groups in the late stages of storage (7–11 months) increased to 57.1, 56.5, and 44.2%, respectively. The relative abundance of *Aspergillus* in maize kernels from the CC group during the whole storage was obviously lower than the CK and PA groups. The result suggested that the complex essential oils inhibited the growth of toxigenic *Aspergillus*. This confirmed the findings of our and other investigators’ studies (Velluti et al., 2003, 2004; Marín et al., 2004; Bluma and Etcheverry, 2008; čvek et al., 2010; Passone et al., 2012; Yuan et al., 2013). For the CK and CC groups, *Wallemia* also became the main genus in the later stages (7–11 months); the average relative abundance of *Wallemia* in the CK, PA, and CC samples was 15.8, 2.68, and 21.99%, respectively. *Wallemia* is a genus of xerophilic fungi and grows well on substrates with low *a*_w_ (Xing et al., 2016). In previous studies, *Wallemia* was identified in peanuts grown in China (Xing et al., 2016). In the present study, this genera was confirmed to grow well on maize kernels with low *a*_w_ (<0.70) during storage. The relative abundance of *Wallemia* in PA group was lower than CK and CC group with the concomitant increase in *Fusarium* (including *Gibberella*). The result suggests that propionic acid can highly inhibit the growth of *Wallemia*. However, the inhibitory effect of propionic acid on the growth of *Fusarium* is weaker than that of the essential oils.

In the stored maize, six mycotoxins including AFB_1_, ZEN, DON, FB_1_, OTA, and T-2 were detected. Of them, AFB_1_, ZEN, and DON were the major mycotoxins presented in maize kernels during storage and this result is consistent with those reported in previous studies. Mudili et al. (2014) found that AFB_1_, FB_1_, T-2, DON, and OTA were the main mycotoxins in freshly harvested maize kernels from India with the ranges of concentration 48–58, 76–123, 38–50, 72–94, and <5 μg/kg, respectively. Rocha et al. (2009) investigated the co-occurrence of fumonisins (FUM) and AFs in freshly harvested corn grains from four regions of Brazil and observed that 98% of corn grains were contaminated with FB_1_ and 74.5% with FB_1_ and FB_2_, with levels ranging 0.02–9.67 μg/kg for FB_1_ and 0.02–3.16 μg/kg for FB_2_. Twenty-one (10.5%) corn samples were contaminated with AFB_1_, 7 (3.5%) with AFB_2_, and only 1 (0.5%) with AFG_1_ + AFG_2_. Co-occurrence of FUM and AFs was observed in 7% of corn samples. Chauhan et al. (2016) found that the mean AFs concentration of maize samples collected from the Gedeo zone of Ethiopia was 53 μg/kg. Of 150 samples, 80 (53%) samples possessed >50 μg/kg of AFs and 57 (38%) had 40–50 μg/kg of AFs. Czembor et al. (2015) reported that DON, ZEN, and FB_1_ were the main mycotoxins in maize grain grown in Poland. FB_1_ was detected in all tested samples, and DON and ZEN were found in 66.67 and 43.33% of samples, respectively. Xing et al. (2017a) showed that FB_1_ and DON were the main toxins in maize kernels from the North China Plain, followed by ZEN and AFB_1_. All maize kernels were contaminated with FB_1_ with concentrations ranging 16.5–315.9 μg/kg. All the post-nature drying maize were contaminated with DON ranging 5.8–9843.3 μg/kg, while 7 of 22 pre-nature drying samples were contaminated with 50.7–776.6 μg/kg of DON (Xing et al., 2017a).

**FIGURE 5:**
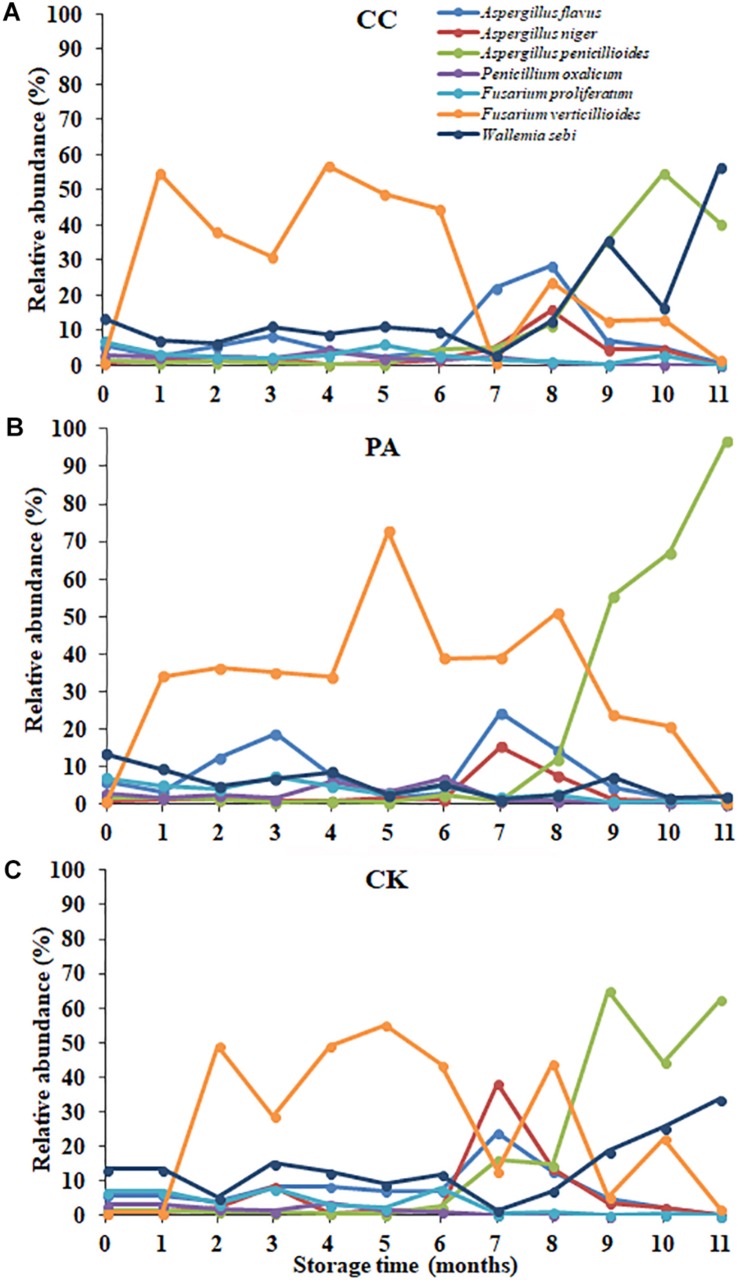
The relative abundance of predominant taxonomic groups at species level over storage time in stored peanuts. **(C)** CK, control group; **(B)** PA, propionic acid group; **(A)** CC, complex essential oils group.

In maize kernels, AFB_1_ is mainly produced by *A. flavus* and *A. parasiticus*. In the present study, *A. flavus* was the main species with the relative abundances of 7.78, 9.27, and 9.13% in CK, PA, and CC group, respectively. For the CK group, the relative abundance of *A. flavus* and the content of AFB_1_ at 7–8 months were both significantly higher than that at other months. ZEN is mainly biosynthesized by some *Fusarium* species like *F. graminearum*, *Fusarium culmorum*, *Fusarium cerealis*, *Fusarium equiseti*, *F. verticillioides* (*G. fujikuroi*), and *Fusarium incarnatum*. In the present study, *F. verticillioides* (*G. fujikuroi*) was the most predominant species with the relative abundances 30.67, 27.29, and 33.73% in CK, PA, and CC group, respectively (Figure 5). The relative abundance of *F. graminearum* in the three groups was very low. However, DON and ZEN were detected in all maize samples during storage, and especially the contents of DON increased from 0 to 8th month and were high at 8–9 months for the CK group (Table 2). The occurrence of DON and ZEN in the maize suggested that *F. graminearum* (or perhaps *F. culmorum*) should be present in the kernels. Similar findings were observed by Czembor et al. (2015) who found that the incidence of *F. graminearum* was very low in all maize grains grown in Poland while DON and ZEN were detected in the grains. Reid et al. (1999) indicated that *F. verticillioides* germinated and grew over a wider range of *a*_w_ and temperatures than *F. graminearum* (Czembor et al., 2015). The minimum temperatures required for *F. verticillioides* and *F. graminearum* growth are 4 and 10°C, respectively (Cook and Christensen, 1976). Therefore, *F. verticillioides* has the chance to ultimately surpass *F. graminearum* (Czembor et al., 2015). FB_1_ is mainly produced by *F. verticillioides* and *F. proliferatum* (Xing et al., 2014). In the present study, these two fungal species both were main contaminants in stored maize. During storage, the relative abundances of *F. proliferatum* in the CK, PA, and CC groups were 3.04, 3.00, and 2.24%, respectively. The content of FB_1_ in the CC group was lower than that in the CK and PA groups due to the lower total fungal counts and the lower relative abundance of *F. proliferatum*. These results suggest that there is a high correlation between the occurrence of mycotoxin-producing fungi and corresponding mycotoxins.

## Conclusion

To evaluate the application prospect of the complex essential oils in practical stored maize during 12 months of storage, the inhibition efficiency of complex essential oils on fungal growth and mycotoxins production was evaluated using plate counting, ITS2 sequencing, and LC–MS/MS. To our knowledges, this is the first study that provides a snapshot of the fungal microbiome in stored maize, and some solid evidences for the practical application of essential oils in grains during storage. In general, *Aspergillus*, *Fusarium*, *Wallemia*, *Sarocladium*, and *Penicillium* were main genera in maize and the fungal diversity decreased over storage time. At the early and later stages of storage, *Fusarium* and *Aspergillus* were predominant genera, respectively. Results from our study confirmed critical information that the complex essential oils (0.02%) highly reduced the total fungal counts, the contents of AFB_1_, ZEN, and DON in stored maize, and was more effective than propionic acid (0.2%). In particular, the essential oils had stronger inhibitory effect on toxigenic *Aspergillus*, while propionic acid was more effective against *Wallemia*. These findings provide substantial solid evidence for the successful application of the complex essential oils in controlling toxigenic fungi and subsequent mycotoxins contamination during storage.

## Data Availability

Publicly available datasets were analyzed in this study. This data can be found here: https://www.ncbi.nlm.nih.gov/sra/PRJNA542593 and https://www.ncbi.nlm.nih.gov/sra/PRJNA542593.

## Author Contributions

FX and YL conceived and designed the experiments. LW, BL, JJ, LM, XD, LP, and YZ performed the experiments. FX and LW analyzed the data and wrote the manuscript.

## Conflict of Interest Statement

The authors declare that the research was conducted in the absence of any commercial or financial relationships that could be construed as a potential conflict of interest.

## Footnotes

1 http://faostat.fao.org

2 http://data.stats.gov.cn/easyquery.htm?cn=C01
